# NINJ1 in Cell Death and Ferroptosis: Implications for Tumor Invasion and Metastasis

**DOI:** 10.3390/cancers17050800

**Published:** 2025-02-26

**Authors:** Ssu-Yu Chen, Ing-Luen Shyu, Jen-Tsan Chi

**Affiliations:** 1 Department of Pharmacology and Cancer Biology, Duke University School of Medicine, Durham, NC 27710, USA; ssu-yu.chen@duke.edu; 2 Department of Molecular Genetics and Microbiology, Duke University School of Medicine, Durham, NC 27710, USA; 3 Department of Obstetrics and Gynecology, Chi Mei Medical Center, Tainan 710, Taiwan; 4 Department of Pharmacy, Chia Nan University of Pharmacy and Science, Tainan 717, Taiwan; 5 Center for Advanced Genomic Technologies, Duke University School of Medicine, Durham, NC 27710, USA

**Keywords:** NINJ1, ferroptosis, plasma membrane rupture, tumor invasion, metastasis

## Abstract

NINJ1 was initially recognized for its role in nerve regeneration and cellular adhesion. Subsequent studies have uncovered its participation in cancer progression, where NINJ1 regulates critical steps in tumor metastasis, such as cell migration and invasion. More recently, NINJ1 has emerged as a multifunctional protein mediating plasma membrane rupture (PMR) in several lytic cell death processes, including apoptosis, necroptosis, and pyroptosis. However, its role in ferroptosis—an iron-dependent form of lytic cell death characterized by lipid peroxidation—remained unclear until 2024. Ferroptosis is a tumor suppression mechanism that may be particularly relevant to detached and metastatic cancer cells. This review explores the role of NINJ1 in tumor invasion and metastasis, focusing on its regulation of ferroptosis via a non-canonical mechanism distinct from other cell deaths. We discuss the process of ferroptosis and its implications for cancer invasion and metastasis. Furthermore, we review recent studies highlighting the diverse roles of NINJ1 in ferroptosis regulation, including its canonical function in PMR and its non-canonical function of modulating intracellular levels of glutathione (GSH) and coenzyme A (CoA) via interaction with xCT anti-porter. Given that ferroptosis has been associated with tumor suppression, metastasis, the elimination of treatment-resistant cancer cells, and tumor dormancy, NINJ1′s modulation of ferroptosis presents a promising therapeutic target for inhibiting metastasis. Understanding the dual role of NINJ1 in promoting or restraining ferroptosis depending on cellular context could open avenues for novel anti-cancer strategies to enhance ferroptotic vulnerability in metastatic tumors.

## Simple Summary

This review summarizes the newly appreciated role of NINJ1 in regulating cell deaths and ferroptosis. It examines how NINJ1 influences ferroptosis through its canonical function in regulating plasma membrane rupture and its non-canonical role in modulating glutathione and coenzyme A levels via its interaction with the xCT anti-porter. The review also discusses the context- and cell type-dependent functions of NINJ1 in various cancer types. Additionally, it highlights the therapeutic potential of activating or inhibiting NINJ1 to affect tumor growth and ferroptosis sensitivity, a key tumor-suppressing mechanism. Understanding how NINJ1 can either promote or suppress ferroptosis, depending on the cancer type, opens possibilities for novel treatments, particularly for preventing metastasis and targeting treatment-resistant cells. This review offers valuable insights for developing innovative anti-cancer strategies.

## 1. NINJ1 and Its Various Biological Functions

### 1.1. Introduction of NINJ1 and Its Role in the Nervous System

**Figure 1:**
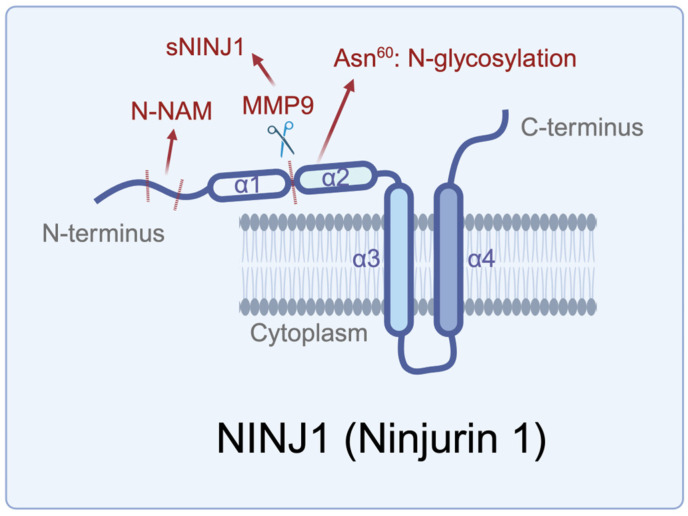
The protein domain structures of NINJ1. Several key locations of modifications and cleavage are indicated.

**Figure 2:**
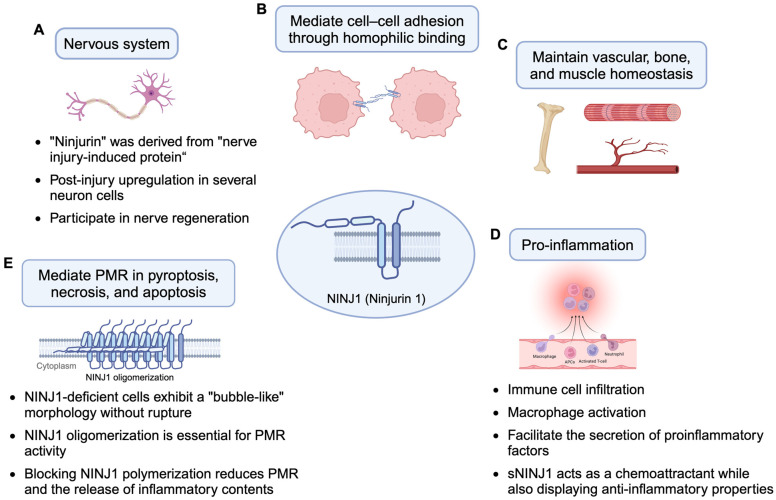
The functions of NINJ1 in various biological contexts. These functions include the nervous system (**A**) in which NINJ1 was first discovered. Subsequent studies identified its role in cell–cell adhesion through homophilic binding (**B**) and in maintaining tissue homeostasis in the vascular system, bone, and muscle (**C**). Additional studies have revealed a prominent role in inflammation by regulating immune cell filtration and activation, as well as the secretion of pro-inflammatory factors (**D**). (**E**) More recently, several studies have shown that NINJ1 mediates plasma membrane rupture (PMR) during multiple forms of regulated cell death, including pyroptosis, necrosis, and apoptosis. Further details are discussed in the main text.

NINJ1 (Ninjurin 1 or nerve injury-induced protein) was first identified in 1996 based on its post-injury upregulation in murine Schwann cells and dorsal root ganglion neurons [1]. Mice lacking NINJ1 display neuropsychiatric-like behaviors, including repetitive and anxiety-driven actions, emphasizing its vital role in neural development (Figure 1 and Figure 2A, Table 1) [2]. NINJ1 encodes a 152-amino-acid double-transmembrane protein (~16.3 kDa) consisting of two extracellular regions at the N- and C-termini, two hydrophobic transmembrane domains, and an intracellular region (Figure 1) [1]. The extracellular α-helices (α1 and α2) are located at the N-termini, while α3 and α4 are embedded within the membrane [3]. The N-terminal extracellular region contains a critical adhesion motif (N-NAM, Pro^26^–Asn^37^) that mediates homophilic cell adhesion, which is essential for nerve regeneration, cellular migration, and communication (Figure 2B) [4]. This region also features an N-glycosylation site at Asn^60^, a posttranslational modification required for NINJ1 to form homologous protein complexes [5]. Additionally, matrix metalloproteinase 9 (MMP9) has been shown to cleave the N-terminal extracellular region between Leu^56^ and Leu^57^, generating a soluble form of NINJ1 (sNINJ1) (Figure 1) [6]. sNINJ1-mimetic peptides, containing the N-NAM sequence, have been shown to promote angiogenesis in human umbilical vein endothelial cells (HUVECs) and the post-ischemic brain by blocking NINJ1 function [7]. These peptides also exhibit anti-inflammatory properties, reducing atherosclerosis in mouse models (Table 1) [8].

### 1.2. The Role of NINJ1 in Maintaining Vascular, Bone, and Muscle Homeostasis

Beyond the nervous system, NINJ1 is also expressed in various embryonic and adult tissues, primarily in epithelial cells, where it supports tissue development and function [4]. It is also critical in tissue homeostasis, particularly in vascular, bone, and muscle systems (Figure 2C, Table 1) [39]. In vascular homeostasis, NINJ1 regulates angiogenesis by modulating Angiopoietin-1 (Ang1) and Angiopoietin-2 (Ang2) expression. During early ocular development, transient NINJ1 upregulation in macrophages enhances Ang2 and Wnt family member 7b (Wnt7b) expression, leading to vascular endothelial cell apoptosis and the regression of the hyaloid vascular system, essential for lens maturation [40]. NINJ1 is also a potential therapeutic target for erectile dysfunction caused by endothelial dysfunction and peripheral neuropathy, as its inhibition promotes penile angiogenesis and neural regeneration via Ang1 and Ang2 signaling, restoring erectile function in diabetic and cavernous nerve injury mouse models [22,23]. In bone homeostasis, NINJ1 promotes the survival of prefusion osteoclasts by reducing caspase-9-dependent apoptosis, influencing bone development [24]. In muscle homeostasis, NINJ1 is upregulated in cardiomyocytes during pathological cardiac hypertrophy. Its N-glycosylated form regulates myogenic differentiation and growth, while the non-glycosylated form is associated with myocyte migration and fusion [25].

### 1.3. The Role of NINJ1 in Inflammation

NINJ1 also plays a critical role in inflammatory conditions, including multiple sclerosis, experimental autoimmune encephalomyelitis, and ischemic stroke [39]. In response to inflammatory stimuli, its expression is significantly upregulated on activated immune cells, such as macrophages, neutrophils, and T lymphocytes. NINJ1 facilitates immune cell migration to sites of inflammation [26,27,28,29,30] and promotes inflammation through Toll-like receptor 4 (TLR4) signaling activation, as demonstrated in a sepsis mouse model [31]. It also contributes to macrophage activation and the secretion of proinflammatory factors, including IL-1β, IL-6, and TGF-β1, in colitis and pulmonary fibrosis [32,33]. Therapeutic interventions targeting NINJ1—such as intranasal siRNA delivery or peptides targeting its N-NAM—have shown anti-inflammatory effects in models of ischemic stroke and systemic inflammation [31,34]. Furthermore, NINJ1 directly binds to the lipid moiety of lipopolysaccharides (LPS) through its transmembrane domain (amino acids 81–100), regulating LPS-induced inflammation [41]. sNINJ1 may act as a chemoattractant due to its structural similarity to chemokines [6], yet it also exhibits anti-inflammatory properties, such as reducing atherosclerosis [8], highlighting its complex and context-dependent roles in inflammation (Figure 2D, Table 1).

## 2. The Role of NINJ1 in Mediating the Plasma Membrane Rupture of Different Cell Death

### 2.1. NINJ1 Mediates the Plasma Membrane Rupture in Different Types of Lytic Cell Death

Recent studies have revealed a novel role of NINJ1 in regulated cell death [42]. Plasma membrane rupture (PMR), traditionally considered a passive, osmotic pressure-driven event, is the final stage of various forms of regulated cell death. PMR facilitates the release of damage-associated molecular patterns (DAMPs) that amplify inflammatory responses. Using a forward-genetic screen on bone marrow-derived macrophages (BMDMs) from randomly mutagenized mice, researchers found that mutations or a complete knockout of *NINJ1* effectively blocked PMR during pyroptosis, apoptosis, or necrosis. NINJ1-deficient cells displayed a distinct “bubble-like” morphology without rupturing despite being non-viable, as confirmed by ATP production measurements. Additionally, these cells exhibited a reduced release of intracellular proteins, including high mobility group box 1 (HMGB1), a well-known DAMP, and lactate dehydrogenase (LDH), a standard PMR marker. In a separate study, glycine has been shown to protect various cells against cell death by inhibiting NINJ1 [43]. These findings highlight the critical role of NINJ1 in the last stage of PMR of multiple forms of regulated cell death and its importance in driving inflammatory responses through the release of HMGB1 and other cellular contents.

### 2.2. Proposed Structural Models of How NINJ1 Mediates Plasma Membrane Rupture

Several structural models have been proposed to explain how NINJ1 mediates PMR. First, NINJ1 oligomerization, driven by its extracellular α-helices, is essential for PMR activity [42]. In its inactive state, NINJ1 forms a face-to-face homodimer with a three-helix conformation and an unkinked transmembrane helix one, which maintains the autoinhibition of PMR. Activation requires the disruption of this dimerization, transitioning NINJ1 into a four-helix kinked conformation [44]. This activation occurs in two steps: oligomer formation, followed by functionalization into lesions, dependent on cell swelling rather than intracellular pressure [45]. Two opposing mechanisms have been proposed to explain how activated NINJ1 mediates PMR. The pore-forming model suggests that extracellular helices α1 and α2 insert into the membrane, forming filaments that stabilize lesions and release intracellular contents, such as LDH and DAMPs. Such a mechanism resembles the action of gasdermin D [3]. In contrast, the “cookie-cutter” model proposes that NINJ1 forms hydrophilic rings that excise membrane patches, leading to fragmentation and eventual PMR [46]. Key residues, including glycine at transmembrane kinks and positively charged residues near the inner leaflet of the plasma membrane, are critical for proper NINJ1 localization [46]. Despite these findings, the exact signals triggering NINJ1 oligomerization to mediate PMR remain unknown.

### 2.3. Therapeutic Potential of NINJ1 in Inflammatory Disease Through Regulating Plasma Membrane Rupture

Based on such a novel function in cell death, the genetic and chemical manipulation of NINJ1 shows significant therapeutic potential for various inflammatory diseases. In mouse models of liver damage—including ischemia–reperfusion injury, anti-Fas antibody treatment, and concanavalin A injection—blocking NINJ1 polymerization with a monoclonal antibody or through genetic deficiency significantly reduced PMR and the release of inflammatory contents, thereby alleviating tissue inflammation and liver pathology [35]. Glycine is known for its cytoprotective effects, inhibiting NINJ1 membrane clustering and preventing PMR-related damage [43]. In severe acute pancreatitis, NINJ1 expression is upregulated via Ca^2^⁺ signaling and the p53/NINJ1 pathway, and the *NINJ1* knockout delays PMR in acinar cells, mitigating disease progression [36]. Similarly, in acute oxalate nephropathy, myeloid-specific *NINJ1* deletion alleviated kidney injury by reducing HMGB1 release and neutrophil extracellular trap formation [37]. Furthermore, NINJ1-dependent PMR facilitates the release of procoagulant microvesicles during pyroptosis, driving systemic coagulation and inflammation. Inhibiting NINJ1 through haploinsufficiency or glycine treatment reduces cytokine release and protects against sepsis-induced coagulopathy [38]. These findings underscore NINJ1 as a compelling therapeutic target for controlling inflammation and tissue damage associated with cell death (Figure 2E, Table 1).

## 3. Ferroptosis—A Newly Recognized Form of Regulated Cell Death

Ferroptosis is a recently recognized regulated cell death driven by iron accumulation and lipid peroxidation [47]. Ferroptosis is also a lytic type of cell death. Although distinct from apoptosis, necrosis, and autophagy in its morphology, biochemistry, and genetics, ferroptosis shares several necrosis-like morphological features [48]. These include moderate chromatin condensation, cytoplasmic swelling (oncosis), organelle swelling, and PMR. Additional characteristics involve increased autophagosome formation, mitochondrial abnormalities, cell rounding, and detachment [49]. Remarkably, ferroptosis can propagate rapidly to neighboring cells, resulting in a wave-like spread of cell death [50]. Below, we will summarize several important regulatory mechanisms that are important in ferroptosis.

### 3.1. Iron Homeostasis

**Figure 3:**
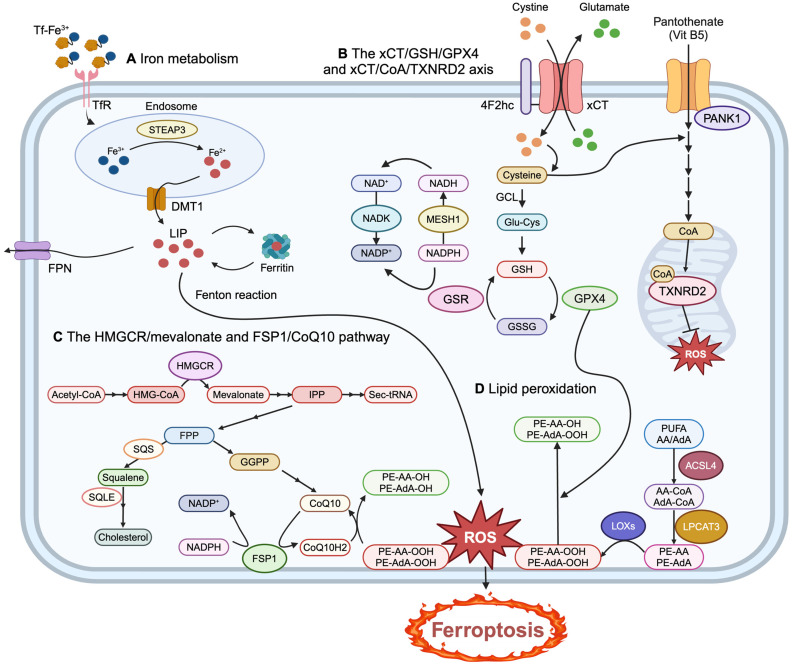
Summary of ferroptosis mechanism. These mechanisms include (**A**) the regulation of iron metabolism that promotes iron-driven Fenton amplification. (**B**) The endogenous ferroptosis-protecting metabolites, including the synthesis and downstream mediators of GSH- and CoA-mediated ferroptosis protection. (**C**) Metabolic pathways of the HMGCR/mevalonate and FSP1/CoA10 pathways. (**D**) The abundance of different ferroptosis-promoting and ferroptosis-inhibiting lipid species that reprogram membrane susceptibility to lipid peroxidation. Further details are discussed in the main text.

Iron homeostasis is tightly regulated at cellular and systemic levels to ensure sufficient iron for essential biological functions while preventing overload and toxicity. Iron exists in two forms: ferrous (Fe^2+^) and ferric (Fe^3+^) and is crucial for synthesizing metalloproteins and cofactors like heme and Fe/S clusters. After absorption in the gut, Fe^3+^ binds to transferrin (Tf) in the plasma and is delivered into cells via the transferrin receptor (TfR), which is internalized through the endosomal pathway. In the endosome, the six-transmembrane epithelial antigen of prostate 3 (STEAP3) reduces Fe^3+^ to Fe^2+^, allowing divalent metal transporter 1 (DMT1) to export the iron. The iron is either stored in ferritin or exported by ferroportin (FPN) to maintain safe levels of labile iron [51]. Dysregulated iron metabolism, such as increased iron absorption or impaired storage and export, can trigger ferroptosis through two primary mechanisms: iron-mediated reactive oxygen species (ROS) production via the Fenton reaction and the activation of iron-containing enzymes like lipoxygenases (LOXs). Iron accumulation also promotes lipid peroxidation, especially in polyunsaturated fatty acids (PUFAs), leading to membrane damage and rupture [52]. The regulation of key iron metabolism genes—such as ferritin heavy chain (FTH1), ferritin light chain (FTL), Tf, TfR, FPN, and DMT1—also influences sensitivity to ferroptosis (Figure 3A) [53,54,55,56].

### 3.2. Lipid Peroxidation

Fatty acids are essential for cellular functions such as energy production, membrane formation, and signaling. They are classified into saturated, monounsaturated (MUFAs), and polyunsaturated (PUFAs), with PUFA peroxidation being particularly important in ferroptosis. Ferroptotic cell death is driven by the peroxidation of membrane phospholipids containing PUFAs, not free PUFAs. Enzymes such as acyl-CoA synthetase long chain family member 4 (ACSL4) and lysophosphatidylcholine acyltransferase 3 (LPCAT3) are essential for incorporating PUFAs into phospholipids, facilitating their integration into the plasma membrane and regulating ferroptosis. Among membrane phospholipids, arachidonic acid (AA) and adrenic acid (AdA)-containing phosphatidylethanolamine (PE) and phosphatidylcholine (PC) are primary targets for lipid peroxidation. This process occurs through non-enzymatic autoxidation, which requires ROS, especially hydroxyl radicals (OH·), from the Fenton reaction and enzyme-mediated processes catalyzed by LOXs (Figure 3D). Linoleic acid (LA) and AA are common LOX substrates. Other enzymes, such as NADPH oxidases (NOXs) and cytochrome P450 oxidoreductase (POR), also contribute to ferroptosis [49,51,53,54,55,57]. The resulting lipid peroxides increase membrane tension, activating channels like Piezo1 and transient receptor potential (TRP), which induce non-selective cation influx, cell swelling, and, eventually, membrane rupture [58]. Additionally, lipid peroxides can also decompose into toxic derivatives, such as 4-hydroxynonenal (4-HNE) and malondialdehyde (MDA), which react with DNA, proteins, and other nucleophilic molecules, resulting in significant cytotoxicity [59]. Therefore, lipid peroxidation levels, 4-HNE, and MDA can serve as biomarkers of ferroptosis in both tissues and cells.

### 3.3. Cell Intrinsic and Environmental Protectors of Ferroptosis and Ferroptosis-Inducing Agents

Despite the above-mentioned processes that trigger ferroptosis, various endogenous mechanisms protect cells against lipid damage and ferroptosis. These protective mechanisms include both cell-intrinsic and exogenous factors. Cell-intrinsic systems include the *SLC7A11* (xCT)-glutathione (GSH)-glutathione peroxidase 4 (GPX4) axis, the xCT-coenzyme A (CoA)-mitochondrial thioredoxin reductase 2 (TXNRD2) axis, the 3-hydroxy-3-methylglutaryl-CoA reductase (HMGCR)/mevalonate pathway, and the ferroptosis suppressor protein 1 (FSP1)–Coenzyme Q10 (CoQ10) pathway. The inhibition of these pathways disrupts cellular defense against lipid peroxidation, leading to iron-catalyzed membrane damage, cell rupture, and ferroptosis. Beyond these intrinsic mechanisms, various environmental factors, such as the cellular context, cellular density, lymphatic fluids, and ascites, also contribute to protection against ferroptosis.

Ferroptosis-inducing agents (FINs) can be classified into distinct categories based on their mechanisms of action. Class I FINs inhibit xCT, impairing cystine uptake and GSH synthesis. Class II FINs directly target GPX4 by inhibiting its activity or promoting its degradation. Class III FINs deplete both GPX4 and CoQ10, while Class IV FINs increase the cytosolic labile iron pool (LIP), directly oxidize iron, and indirectly inhibit GPX4 [51,60,61]. The following sections will introduce the key intrinsic antioxidant pathways and proteins involved in ferroptosis regulation and the mechanisms through which different FINs exert their effects (Figure 3).

#### 3.3.1. xCT Anti-Porter

The system Xc⁻ is a sodium-independent anti-porter that facilitates the export of intracellular glutamate and the import of extracellular cystine in a 1:1 ratio. It consists of two subunits: solute carrier family 7 member 11 (*SLC7A11*), which encodes the xCT protein responsible for cystine-glutamate transport, and solute carrier family 3 member 2 (*SLC3A2*), which encodes the CD98 (or 4F2hc) protein, stabilizing xCT and ensuring its proper membrane localization [62]. GSH, the most abundant cellular antioxidant and a critical cofactor for GPX4, requires cysteine, sulfur-containing amino acids, and the enzymatic activity of glutamate–cysteine ligase (GCL) for its synthesis [49]. Although cysteine can be produced through de novo biosynthesis via the transsulfuration pathway or through protein degradation, most cancer cells predominantly depend on xCT-mediated cystine uptake, which is then reduced to cysteine in an NADPH-dependent reaction [63]. GSH regeneration, in turn, requires the enzymatic activity of glutathione reductase (GSR), which also catalyzes its reduction in an NADPH-dependent manner (Figure 3B).

Metazoan SpoT homolog 1 (MESH1), the metazoan homolog of bacterial SpoT—a regulator of the bacterial stringent response—was recently identified as the first mammalian cytosolic NADPH phosphatase, playing a critical role in regulating ferroptosis sensitivity. Our group discovered that silencing MESH1 alleviates NADPH depletion during ferroptosis, resulting in increased levels of reduced GSH and enhanced protection against ferroptosis. However, this protective effect was abolished by the simultaneous silencing of NADK, an NAD kinase essential for NADPH production. These findings highlight the pivotal role of MESH1 and its NADPH enzymatic activity in modulating oxidative stress and ferroptosis (Figure 3B) [64,65].

In addition to its role in regulating GSH, the xCT transporter has also been implicated in CoA biosynthesis, which requires cysteine, pantothenate, and ATP [66]. CoA supplementation specifically prevents ferroptosis induced by xCT inhibitors but not GPX4 inhibitors [67,68,69]. The ferroptosis-protective effect of CoA is mediated through the regulation of mitochondrial TXNRD2 enzymatic activity. CoA achieves this by covalently modifying the thiol group of cysteine on Cys-483 via a process known as CoAlation. Since the thioredoxin system operates alongside the GSH system to maintain cellular redox balance and protect against ferroptosis, the modulation of CoA levels and TXNRD2 enzymatic activity can influence ferroptosis sensitivity (Figure 3B) [16,68].

Class I FINs promote ferroptosis by depleting GSH, thereby indirectly inhibiting GPX4 activity. This can be achieved through various mechanisms, such as targeting xCT to reduce cystine uptake (e.g., using erastin, imidazole ketone erastin (IKE), and sulfasalazine (SAS)), directly depleting cysteine with engineered cyst(e)inase [70], or inhibiting GSH synthesis with compounds like buthionine sulfoximine (BSO), which blocks GCL activity [71]. Furthermore, the inhibition of xCT or cysteine depletion not only disrupts GPX4 activity but also impairs other critical pathways, including CoA synthesis [16,68], which may influence cellular sensitivity to ferroptosis.

#### 3.3.2. Glutathione Peroxidase 4 (GPX4)

GPX4, originally called phospholipid hydroperoxide GPX (PHGPX4), is a unique enzyme within the glutathione peroxidase family containing selenocysteine. It plays a pivotal role in protecting cells from ferroptosis by reducing lipid hydroperoxides (PLOOH) to lipid alcohols (PLOH), thereby mitigating lipid peroxidation. GPX4 is a multifunctional enzyme capable of reducing peroxidized lipids in various forms: free lipids, lipids complexed with phospholipids (PLs) or proteins like lipoproteins, and those embedded within membranes. Unlike other glutathione peroxidases, GPX4 functions as a monomer and employs a ping-pong catalytic mechanism. In this process, its active selenol group (-SeH) is oxidized by peroxides to selenic acid (-SeOH), reduced by GSH to form a selenylsulfide intermediate (-Se-SG), and subsequently regenerated by a second GSH molecule, yielding glutathione disulfide (GSSG). Thus, GPX4 activity critically depends on both selenocysteine and GSH (Figure 3B) [49,54,61].

Class II FINs interfere with GPX4 activity directly or promote its degradation. Notable small-molecule inhibitors include RAS-selective lethal 3 (RSL3), which inactivates GPX4 by alkylating its selenocysteine residue, DPI7 (ML162) and DPI10 (ML210), which inhibits GPX4 through covalent binding, and altretamine, which directly inhibits GPX4 [61,72,73,74].

#### 3.3.3. The HMGCR/Mevalonate Pathway

The mevalonate pathway, regulated by 3-hydroxy-3-methylglutaryl-CoA reductase (HMGCR), is critical in ferroptosis by producing isopentenyl pyrophosphate (IPP) which is required for the synthesis of Sec-tRNA that incorporates selenocysteine into selenoproteins like GPX4. Additionally, IPP contributes to the synthesis of ubiquinone (CoQ10) through a series of enzymatic steps, including the formation of key intermediates such as farnesyl diphosphate (FPP) and geranylgeranyl diphosphate (GGPP). CoQ10 is subsequently reduced to ubiquinol (CoQ10H2) by FSP1, producing a lipophilic antioxidant that protects against lipid peroxidation and ferroptosis (Figure 3C) [53,54,55,75,76]. The inhibition of HMGCR by statins suppresses the mevalonate pathway and GPX4 expression, promoting ferroptosis in several cancer cells [77,78]. Similarly, the antimalarial drug artesunate inhibits the nuclear translocation of sterol regulatory element binding transcription factor 2 (SREBP2), reducing *HMGCR* transcription, IPP levels, and GPX4 synthesis, thereby triggering ferroptosis. Interestingly, SREBP2 also regulates Tf expression, which decreases intracellular iron levels and mitigates lipid peroxidation and ferroptosis [79,80,81].

Downstream enzymes and intermediates of the mevalonate pathway further regulate ferroptosis. FPP, a derivative of IPP, is critical for CoQ10 synthesis, which supports ferroptosis regulation. Squalene synthase (SQS) converts FPP to squalene, while squalene epoxidase (SQLE) processes squalene into cholesterol (Figure 3C) [55,81]. The activation of SQS by FIN56, a class III FIN, suppresses Sec-tRNA prenylation and CoQ10 synthesis, leading to lipid peroxidation and ferroptosis [82]. Conversely, squalene itself has been reported to suppress ferroptosis by increasing GSH levels, reducing Fe^2^⁺, and regulating ferroptosis-related genes, such as *SLC7A11* and *ACSL4* [83]. Furthermore, SQLE has been shown to confer ferroptosis resistance in breast cancer models by reducing intracellular ROS levels [84].

#### 3.3.4. The FSP1–CoQ10 Pathway

Ferroptosis suppressor protein-1 (FSP1), also known as apoptosis-inducing factor mitochondria-associated 2 (AIFM2), is a key ferroptosis regulator that operates independently of the GSH–GPX4 pathway. As a NADPH-dependent oxidoreductase, FSP1 converts CoQ10 into its reduced antioxidant form, CoQ10H2, which halts lipid peroxidation and protects cells from ferroptosis. FSP1 is localized to the plasma membrane and organelle-enriched membranes, such as the endoplasmic reticulum and Golgi apparatus, via an N-myristoylation signal. Notably, FSP1 specifically requires NADPH (not NADH) for its ferroptosis-suppressing function and is regulated by the Nrf2–Keap1 pathway, underscoring its therapeutic potential as a target in cancers resistant to ferroptosis [49,53,55]. Beyond its CoQ10-dependent antioxidant role, FSP1 mediates ferroptosis resistance through nonclassical redox cycling of vitamin K, producing vitamin K hydroquinone (VKH2), another potent scavenger of lipid peroxides [85]. Additionally, FSP1 protects against ferroptosis via mechanisms involving the endosomal sorting complexes required for the transport III (ESCRT-III) complex, which plays a critical role in repairing damaged plasma membranes during ferroptosis or other forms of cell death. This process, involving key components such as CHMP5 and CHMP6, is independent of CoQ10H2 activity [86,87,88].

CoQ10, the main effector of the FSP1 pathway, is a vital lipid-soluble antioxidant and redox carrier widely distributed across mammalian cell membranes, with particularly high concentrations in the heart, liver, kidney, and pancreas. It has two forms: oxidized (ubiquinone, CoQ10) and reduced (ubiquinol, CoQ10H2). In addition to its role in mitochondrial respiratory function, CoQ10 neutralizes free radicals in non-mitochondrial membranes and facilitates electron transport in the plasma membrane and Golgi apparatus, further supporting ferroptosis resistance [52,53,54]. Supplementation with CoQ10 or its hydrophilic analog, idebenone, has demonstrated protective effects against ferroptosis, whereas inhibiting CoQ10 biosynthesis accelerates ferroptotic cell death [82].

Class III FINs primarily enhance ferroptosis sensitivity by downregulating mevalonate-derived CoQ10. FIN56 activates SQS, suppressing Sec-tRNA prenylation and CoQ10 synthesis, leading to lipid peroxidation and ferroptosis. Additionally, first-generation (iFSP1) and second-generation (icFSP1) inhibitors of FSP1 have been shown to induce ferroptosis independently of GPX4 activity. Furthermore, by inhibiting HMGCR and the mevalonate pathway, various statins block CoQ10 biosynthesis, reduce GPX4 expression, and promote ferroptosis [60,89,90].

## 4. The Role of Ferroptosis in Tumor Metastasis

Ferroptosis has emerged as a critical tumor-suppressing mechanism. Cancer cells accumulate significantly higher iron levels than normal cells [91], requiring an elevated metabolic rate to sustain their rapid proliferation [92]. This increased metabolic demand increases ROS production, thus enhancing reliance on antioxidant defenses to mitigate oxidative stress and avoid cell death. For example, the inappropriate activation of *NRF2* associated with *KEAP1* loss-of-function mutation is an important oncogenic event in many human cancers [93]. Consequently, targeting these antioxidant mechanisms offers a promising therapeutic strategy to induce oxidative stress-driven cell death, such as apoptosis and ferroptosis [94]. Moreover, various biological processes during cancer metastasis render cells more susceptible to ferroptosis [95]. Ferroptosis has also been identified as an effective approach to overcome cancer therapy resistance and contributes to tumor immunity [54]. In the following sections, we will focus on the role of ferroptosis in tumor invasion and metastasis and highlight several key regulators involved in these processes.

### 4.1. Regulation of Ferroptosis by Cell–Cell Contact and YAP/TAZ/Hippo Pathway

The invasion-metastasis cascade is a multi-step process through which cancer cells spread from primary tumors to establish colonies in distant tissues. Such events involve a series of biological processes, termed metastatic cascades, including local invasion, intravasation into the circulatory system, survival during transit in the circulation, extravasation into distant tissues, micrometastatic colony formation, and eventual growth into clinically detectable metastatic lesions [96]. Ferroptosis sensitivity in cancer cells is heavily influenced by cell density and cell–cell contact. Cancer cells grown at low density are more susceptible to ferroptosis, whereas those at high density or in 3D spheres exhibit resistance [97,98,99,100,101,102,103]. This phenomenon may also explain why epithelial-to-mesenchymal transition (EMT), which decreases cell–cell contact, enhances ferroptosis sensitivity, while cancer cells traveling as clusters are protected due to increased cell density [104]. This density-dependent ferroptosis sensitivity is regulated by the Hippo pathway and its effectors, Yes-associated protein 1 (YAP) and transcriptional coactivator with PDZ-binding motif (TAZ). At high cell density, the Hippo pathway is activated, leading to the suppression of YAP and TAZ activity. Specifically, TAZ has been shown to play a critical role in ferroptosis sensitivity in renal and ovarian cancer cells, where its activation at low density promotes ferroptosis, while its suppression at high density confers resistance [97]. Similarly, in breast cancer and hepatic stellate cells involved in liver cirrhosis, YAP serves as a key regulator of density-dependent ferroptosis, with increased cell contact at high density suppressing ferroptosis via YAP inhibition [103,105,106,107]. These findings highlight the central roles of YAP and TAZ as critical regulators of ferroptosis sensitivity, linking cell density and contact to ferroptosis resistance in cancer cells.

### 4.2. Regulation of Ferroptosis by Epithelial-Mesenchymal Transition (EMT)

EMT plays a pivotal role in cancer progression by converting epithelial cells into mesenchymal-like cells with increased motility, invasiveness, and metastatic potential. EMT also contributes to therapy resistance by activating survival pathways and inhibiting apoptosis, making cancer cells more difficult to eliminate [108]. Paradoxically, while EMT confers resistance to many therapies, it simultaneously enhances sensitivity to ferroptosis [95,109]. Several genes associated with EMT modulate ferroptosis sensitivity. For instance, zinc finger E-box binding homeobox 1 (*ZEB1*) promotes the accumulation of polyunsaturated fatty acid-containing phospholipids (PUFA-PL) through peroxisome proliferator-activated receptor gamma (PPARγ) activation, leading to GPX4 dependency and heightened ferroptosis susceptibility [110]. In pancreatic cancer, the glucose-induced O-GlcNAcylation of ZEB1 stabilizes and translocates it to the nucleus, where it activates lipogenesis genes (such as *FASN* and *FADS2*), inducing lipid peroxidation and ferroptosis [111]. Similarly, in head and neck cancer, *ZEB1* overexpression or cadherin 1 (*CDH1*) silencing increases ferroptosis sensitivity [112]. Additionally, the EMT-associated adhesion protein metadherin (MTDH), which drives EMT, invasion, and metastasis, is also linked to ferroptosis. Suppressing MTDH reduces GPX4 and 4F2hc expression, disrupting cysteine and GSH synthesis, thereby enhancing ferroptosis sensitivity [113]. In recurrent breast tumors, the EMT-driven upregulation of discoidin domain receptor tyrosine kinase 2 (*DDR2*) maintains growth advantages while also activating YAP/TAZ-mediated ferroptosis susceptibility [114]. Furthermore, the modulation of the KDM5A–MPC1 pathway increases ferroptosis susceptibility by regulating both EMT and mitochondrial metabolism [115]. On the other hand, iron metabolism also plays a crucial role in ferroptosis sensitivity during EMT. Specifically, the CD44-mediated iron endocytosis pathway is upregulated in EMT, where internalized iron catalyzes the epigenetic activation of mesenchymal proteins, increasing ferroptosis vulnerability [116]. Moreover, histone deacetylase inhibitors elevate intracellular iron levels and reduce FPN expression, further enhancing ferroptosis sensitivity [117]. These findings suggest that ferroptosis-inducing drugs could selectively target multidrug-resistant cancer cells with mesenchymal phenotypes, offering promising strategies to overcome therapy resistance.

### 4.3. Regulation of Ferroptosis by Tumor Microenvironment (TME)

The TME, comprising stromal cells (cellular components) and extracellular matrix (ECM) elements (non-cellular components), plays a pivotal role in promoting cancer cell heterogeneity, clonal evolution, and multidrug resistance, ultimately driving tumor progression and metastasis. Tumor cells, as the central regulators of the TME, manipulate these components through complex signaling networks, reprogramming non-malignant cells to support tumor growth, invasion, and therapy resistance [118]. Notably, ferroptosis has also been implicated in these processes, contributing to antitumor effects within the TME [54,95,109,119].

Ferroptosis has emerged as a potential form of immunogenic cell death (ICD) capable of triggering antitumor immune responses. This process involves the release of DAMPs, such as ATP and HMGB1, which activate the adaptive immune system and promote tumor elimination [120]. Intriguingly, HMGB1 not only acts as a DAMP but also regulates ferroptosis. In NRAS-mutant leukemia cells, HMGB1 deficiency suppresses JNK/p38 signaling, reducing iron uptake, ROS production, and ferroptotic cell death [121]. The immunogenic potential of ferroptosis was further demonstrated in a prophylactic tumor vaccination model, where early ferroptotic cells elicited robust antitumor immunity, whereas late ferroptotic cells lacked this effect, unlike late apoptotic cells [122,123]. Despite its immune-activating potential, ferroptosis can paradoxically contribute to tumor progression by modulating immune cells in the TME. For instance, ferroptotic tumor cells release immunosuppressive factors such as prostaglandin E2 (PGE2) and oxidized lipids, which impair dendritic cell function, antigen presentation, and antitumor immune responses [124,125,126,127]. Additionally, adaptive immunity also intersects with ferroptosis. Cytotoxic CD8^+^ T cells induce ferroptosis in tumor cells by secreting interferon-gamma (IFNγ), which inhibits xCT anti-porter, leading to GSH depletion and lipid peroxidation [128]. However, CD8^+^ T cells in the TME are also vulnerable to ferroptosis due to lipid ROS accumulation, weakening their antitumor efficacy. Strategies targeting CD36, a fatty acid translocase, or inhibiting ferroptosis pathways in T cells have shown promise in restoring immune function and improving therapeutic outcomes [129,130]. These findings underscore ferroptosis as a double-edged sword: while it can activate robust antitumor immunity, it also harbors immunosuppressive effects within the TME, presenting challenges and opportunities for cancer therapy.

Circulating tumor cells (CTCs) spread through the blood vessels or lymphatic system during metastasis, with their microenvironment playing a critical role in their survival and metastatic potential. Cancer cells entering the bloodstream are exposed to high oxidative stress and elevated iron levels, making them particularly susceptible to ferroptosis and limiting their metastatic ability. In contrast, cancer cells traveling via the lymphatic system encounter lymphatic fluid rich in antioxidants and low in iron, providing a protective environment against ferroptosis. Additionally, oleic acid, a MUFA present in lymphatic fluid, has been shown to protect cancer cells from ferroptosis [131]. Mechanistically, MUFAs inhibit ferroptosis by integrating into plasma membranes via ACSL3 activation and serving as substrates for phospholipid remodeling enzymes, such as the membrane bound O-acyltransferase domain containing 1/2 (MBOAT1/2). These enzymes are upregulated by estrogen (ER) or androgen (AR) receptors, highlighting the importance of this pathway in hormone-receptor-positive cancers, such as ER+ breast and AR+ prostate cancers [132,133]. Similarly, our research demonstrates that lipids in ascitic fluid, including oleic acid, shield ovarian cancer cells from ferroptosis during peritoneal dissemination. This protection involves the downregulation of the mitochondrial enzyme 3-hydroxy-3-methylglutaryl-CoA synthase 2 (HMGCS2), which increases lipid droplet accumulation, and the suppression of TfR, reducing cellular labile iron levels. Combining lipid-lowering drugs like bezafibrate with ferroptosis inducers restores ferroptosis sensitivity in metastatic ovarian cancer cells, effectively reducing peritoneal tumor growth [134].

Hypoxia is a critical microenvironmental factor that promotes metastatic progression. Clinically, hypoxia stabilizes the proteins of hypoxia-inducible transcription factors (HIF-1 and HIF-2) to drive the hypoxia gene expression program that increased distant metastasis, poor survival, and treatment resistance in various cancers [135]. Interestingly, hypoxia also influences ferroptosis sensitivity in cancer cells [95,136]. Specifically, HIF-1α protects against ferroptosis by inducing the expression of genes such as *SLC7A11*, *GCLM*, *SHMT2*, and *PHGDH*, which enhance antioxidant defenses by boosting GSH and NADPH production [137,138]. HIF-1α also reduces the levels of ferroptosis-promoting PUFA through increased intracellular lipid droplet storage [139]. In contrast, HIF-2α promotes ferroptosis by activating genes that increase lipid accumulation, iron levels, and oxidative stress, particularly in renal, colon, and glioblastoma cancers [140,141,142]. These findings highlight the complex role of hypoxia and HIFs in regulating ferroptosis.

## 5. The Emerging Role of NINJ1 in Ferroptosis

Ferroptosis, a lytic form of cell death, is also marked by membrane blebbing and ballooning [143]. Following lipid peroxidation, increased membrane tension activates mechanosensitive cation channels, such as Piezo-1 and TRP channels, leading to calcium influx. This influx is identified as an early event and then precedes the uptake of DNA-binding dyes (indicating membrane integrity loss) and LDH release (a PMR marker). Since osmoprotectants can prevent cell lysis and the activation of these channels disrupts monovalent cation gradients (Na^+^, K^+^), causing cell swelling, ferroptotic cell rupture is thought to result from increased osmotic pressure [50,58,144,145]. However, the involvement of NINJ1, a protein that mediates PMR in other forms of cell death, in these processes has only recently begun to be understood. Below, we will discuss the recently emerging evidence for the canonical and non-canonical roles of NINJ1 in regulating ferroptosis.

### 5.1. NINJ1 Mediates Plasma Membrane Rupture in the Late Stage of Ferroptosis

The role of NINJ1 in ferroptosis has recently garnered attention alongside its established involvement in PMR and DAMP release in other forms of cell death [145,146]. In murine embryonic fibroblasts (MEFs), BMDMs, and NIH/3T3 cells, NINJ1 was shown to mediate the release of small and large molecules, such as Sytox Green dye and LDH, specifically in response to ML162, RSL3, and CuOOH treatment. NINJ1 was found to regulate the late stages of ferroptosis, including membrane integrity loss, cell rupture (measured by LDH release), and DAMP release after cell death. However, it was not required for early ferroptotic events like lipid peroxidation, calcium influx, cell swelling, or cell death (measured by ATP levels). Interestingly, osmotic pressure, rather than ROS, triggers NINJ1 oligomerization. However, NINJ1 oligomerization alone does not guarantee NINJ1-dependent PMR, as seen with erastin and FINO2 treatment, which induce oligomerization without affecting PMR. This suggests that NINJ1 oligomerization is an intermediate step that requires additional factors for full activation.

**Figure 4:**
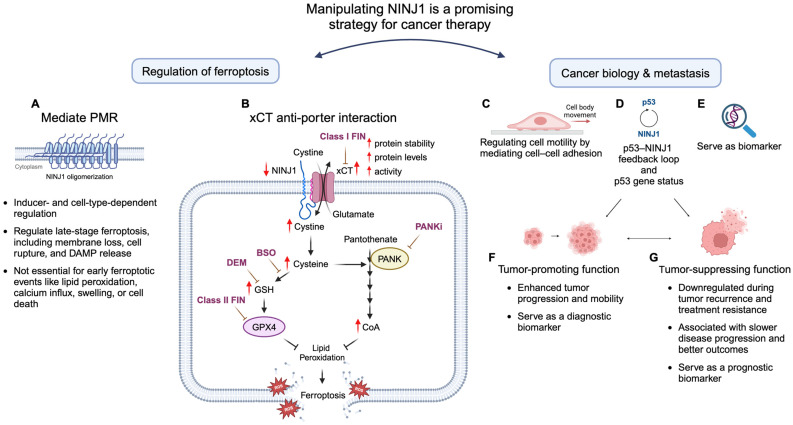
NINJ1 is a potential therapeutic target in cancer treatment by regulating ferroptosis and cancer biology. (**A**) NINJ1 is involved in the very late stage of ferroptosis, including membrane integrity loss, cell rupture (measured by LDH release), and DAMP release after cell death. However, it is not required for earlier ferroptotic events, such as lipid peroxidation, calcium influx, cell swelling, or cell death (measured by ATP levels) [145,146]. (**B**) Non-canonical function of NINJ1 in ferroptosis: NINJ1 influences ferroptosis sensitivity through direct interaction with the xCT anti-porter. NINJ1 knockdown stabilizes xCT, leading to increased cellular levels of GSH and CoA, both essential for ferroptosis protection. Conversely, NINJ1 overexpression destabilizes xCT, reducing GSH and CoA levels and sensitizing cells to ferroptosis induced by xCT inhibitors [16]. (**C**) NINJ1 plays a crucial role in cell motility by mediating cell–cell adhesion through homophilic binding and facilitating immune cell infiltration. This function impacts tumor proliferation, metastasis, and microenvironment [9,10,17,18]. (**D**) NINJ1 has been identified as a transcriptional target of p53, a key tumor suppressor. Knockout or knockdown of *NINJ1* increases p53 expression, potentially by enhancing p53 mRNA translation, suggesting that NINJ1 can also form a feedback loop with p53 [147]. Additionally, the role of NINJ1 in tumorigenesis varies depending on the p53 genetic status [11]. (**E**,**F**) Diagnostic biomarker and therapeutic target: In cancers such as non-small cell lung cancer [12], colitis-associated colorectal cancer [13], hepatocellular carcinoma [14], and acute lymphoblastic B-cell leukemia [15], NINJ1 is overexpressed or associated with cancer progression, making it a valuable diagnostic biomarker. As a result, NINJ1 inhibitors could serve as a potential therapeutic strategy for targeting cancers that depend on NINJ1 for proliferation. (**E**,**G**) Prognostic biomarker and ferroptosis sensitization: In cancers like ovarian cancer [20] and urothelial bladder cancer [21], NINJ1 has been linked to slower disease progression and better patient outcomes, positioning it as a prognostic biomarker. Additionally, since NINJ1 overexpression has been reported to enhance ferroptosis sensitivity [16], NINJ1 activators could be utilized to target cancer cells in these cancer types, as well as in cancers resistant to ferroptosis, while minimizing damage to normal tissues.

However, the role of NINJ1 in ferroptosis appears to be both inducer- and cell-type-dependent. For example, NINJ1 deficiency did not impact PMR induced by erastin or FINO2 and was less critical for RSL3-induced lysis than ML162- or CuOOH-induced lysis. In RAW 264.7 macrophages (a cell line derived from Abelson murine leukemia virus-induced tumors), NINJ1 deficiency provided limited protection against PMR. These findings suggest that the contribution of NINJ1 to PMR varies depending on the intrinsic properties of cell types and the distinct mechanisms of ferroptosis inducers, which differ in how and at what rate they drive cell death. Collectively, these studies reinforce the link between NINJ1-mediated PMR in ferroptosis and its potential role in inflammatory diseases (Figure 4A).

### 5.2. Non-Canonical Role for NINJ1 in Regulating Ferroptosis

In addition to its established role in facilitating PMR during cell death, our recent research has revealed a non-canonical role for NINJ1 in ferroptosis [16]. Specifically, NINJ1 knockdown significantly protected cancer cells from ferroptosis induced by class I FINs (xCT inhibitors) but not by other FINs. This protective effect was validated in several cancer cell types, including fibrosarcoma, triple-negative breast cancer, and ovarian cancer. Through a compound screening approach, we identified CoA and GSH as key players in this protective effect, with both CoA and GSH elevated following NINJ1 knockdown. Furthermore, we uncovered an interaction between NINJ1 and the xCT anti-porter, demonstrating that NINJ1 regulates xCT stability, expression, and function. NINJ1 knockdown enhanced xCT levels and activity by stabilizing the protein, resulting in higher CoA and GSH levels. Both CoA and GSH were essential for ferroptosis protection, as disruption of their synthesis eliminated the observed protective phenotype associated with NINJ1 knockdown. Conversely, NINJ1 overexpression specifically sensitized cells to ferroptosis induced by xCT inhibitors by reducing xCT levels and activity, resulting in decreased CoA and GSH levels. Furthermore, the expression of NINJ1 in the TCGA prostate and colorectal tumors was positively associated with the expression of ferroptosis biomarkers, such as *CHAC1* and *PTGS2*. These findings reveal a novel, non-canonical role for NINJ1 in regulating ferroptosis, distinct from its established function in mediating PMR during lytic cell death. Additionally, the association between NINJ1 and the xCT transporter, along with its impact on GSH and CoA availability, provides insight into a potential mechanism underlying the tumor-suppressive role of NINJ1 in certain cancer types that depend on xCT-mediated metabolic pathways. This study, therefore, highlights the therapeutic potential of manipulating NINJ1 to regulate ferroptosis as a strategy for cancer treatment (Figure 4B).

## 6. The Diverse and Context-Dependent Functional Role of NINJ1 in Cancer

Extensive research highlights the multifaceted role of NINJ1 in cancer biology, with evidence suggesting both tumor-promoting and tumor-suppressing functions. In some studies, elevated NINJ1 levels have been linked to increased tumor progression and mobility, while others indicate that NINJ1 overexpression can suppress tumor growth. Below, we summarize several the key mechanisms through which NINJ1 influences cancer, including its role in modulating cell motility, regulating p53 activity, and its potential as a biomarker (Table 1).

### 6.1. The Role of NINJ1 in Tumor Metastasis and Microenvironment Through Regulating Cell Motility

Cell migration toward favorable environments is a fundamental cellular behavior essential for several key steps in the metastatic cascades, including basement membrane breach, detachment from the primary tumor, intravasation, extravasation, and spread to distant organs [148]. NINJ1 plays a pivotal role in cell motility by mediating cell–cell adhesion through homophilic binding and facilitating immune cell infiltration, thereby influencing tumor proliferation, metastasis, and microenvironment. Notably, NINJ1 deficiency enhances lung cancer cell motility by upregulating ICAM-1 through the IL-6/STAT3 signaling pathway [17], while its overexpression suppresses colitis-mediated colon cancer growth by inhibiting macrophage migration and invasion via the repression of FAK signaling [18]. Conversely, NINJ1 expression is significantly elevated in CTCs from patients with locally advanced prostate cancer, where its overexpression promotes prostate cancer cell migration and invasion in vitro [9]. Additionally, ionizing radiation induces NINJ1 expression in endothelial cells through a p53-dependent mechanism, enhancing endothelial cell and monocyte adhesion and transmigration, contributing to tumor radiotherapy resistance [10]. These findings highlight the multifaceted role of NINJ1 in promoting and inhibiting tumor progression, depending on the cancer type and context (Figure 4C, Table 1).

### 6.2. Regulation of p53 Activity by NINJ1 Through the p53-NINJ1 Feedback Loop

NINJ1 has been identified as a transcriptional target gene of p53, a key tumor suppressor. The knockout or knockdown of *NINJ1* increases p53 expression, potentially by enhancing p53 mRNA translation, suggesting that NINJ1 can also form a feedback loop with p53 [147]. The functional inhibition of NINJ1 suppresses cell proliferation while enhancing apoptosis and premature senescence by activating p53-mediated tumor suppression [147]. Notably, the role of NINJ1 in tumorigenesis varies depending on the p53 genetic status. In cells harboring mutant p53, NINJ1 deficiency promotes cell proliferation and migration, whereas in cells with wild-type p53, it suppresses both cell growth and migration [11]. These findings underscore the dual role of NINJ1 as both a tumor suppressor and promoter, depending on the p53 status. Understanding this context-specific regulation offers promising opportunities for developing targeted cancer therapies by modulating the NINJ1–p53 feedback loop (Figure 4D, Table 1).

### 6.3. Additional Mechanisms of NINJ1 in Cancer and Its Role as a Biomarker

In addition to regulating cell motility and the p53–NINJ1 feedback loop, NINJ1 plays diverse roles in cancer progression and serves as both a diagnostic and prognostic biomarker across various cancer types. For example, NINJ1 has been shown to promote the formation and progression of non-small cell lung cancer (NSCLC) by protecting cancer stem cells from hostile microenvironments via the ligand-independent activation of the LRP6/β-catenin signaling pathway [12]. In a colitis-associated colorectal cancer mouse model, NINJ1 deficiency reduced inflammation and tumor development, with sex-specific effects linked to testosterone, emphasizing the importance of sex differences in its role [13]. Furthermore, NINJ1 is overexpressed in certain cancers, including hepatocellular carcinoma [14] and acute lymphoblastic B-cell leukemia [15], highlighting its diagnostic potential. Conversely, enforced NINJ1 expression has been shown to induce p21 upregulation and cellular senescence independently of p53 in human hepatoma cells [19]. NINJ1 downregulation has been associated with tumor recurrence, treatment resistance, and poorer survival in ovarian cancer patients [20]. Similarly, NINJ1 expression in urothelial bladder cancer has been linked to slower disease progression and better outcomes, underscoring its potential as a prognostic biomarker [21]. These findings illustrate the complex and context-dependent roles of NINJ1 in cancer biology (Figure 4E, Table 1).

## 7. Therapeutic Implications

Given the diverse roles of NINJ1 in cancer biology and ferroptosis, manipulating NINJ1 presents a promising strategy for cancer therapy by simultaneously modulating ferroptosis and tumor invasion. First, as a mediator of PMR, NINJ1 inhibition can reduce the release of pro-inflammatory DAMPs [35], potentially mitigating chronic inflammation and tumor progression [149]. This approach is particularly relevant in cancers with high ferroptosis sensitivity, such as those exhibiting EMT features and activated YAP1/TAZ signaling [97,105,106,107,114,150]. Second, therapeutic strategies that activate NINJ1 could synergize with ferroptosis inducers to enhance therapeutic efficacy. For instance, the xCT anti-porter, often upregulated in cancers, plays a crucial role in tumor cell survival, development, and treatment resistance. Inhibiting xCT has demonstrated significant anti-cancer effects across various cancer types, including lymphoma, leukemia, glioma, prostate cancer, melanoma, lung, ovarian, breast, and pancreatic cancers [151,152]. By regulating xCT stability and ferroptosis sensitivity, combining xCT inhibitors with NINJ1 activation could provide therapeutic benefits in cancers highly dependent on xCT for survival. Third, the interaction of NINJ1 with the TME offers additional opportunities for immunotherapy. For example, enhancing ferroptosis through NINJ1 activation could boost ICD [153], thereby improving immune system engagement against tumors. However, careful modulation is required to avoid releasing immunosuppressive factors, such as oxidized lipids or PGE2, which could dampen antitumor immunity. Fourth, in metastatic settings, strategies aimed at inhibiting NINJ1-mediated motility and adhesion offer a potential strategy to prevent tumor dissemination while sensitizing tumor cells to ferroptosis-inducing agents or other pro-ferroptosis conditions in the bloodstream. Finally, NINJ1 holds therapeutic potential as a biomarker. Its expression levels could predict ferroptosis sensitivity and guide personalized treatment strategies, particularly for cancers resistant to conventional therapies. Future research should focus on refining NINJ1-manipulating agents and exploring their combinatory use with existing treatments to optimize clinical outcomes (Figure 4, Table 1).

## 8. Summary and Future Prospective

In conclusion, NINJ1 is a critical node linking ferroptosis, inflammation, tumor progression, and metastasis, providing a unique therapeutic opportunity to inhibit or treat tumor metastasis. Its dual role in PMR and ferroptosis sensitivity highlights its importance in tumor biology and the TME. While traditionally recognized for its role in PMR during regulated cell death, recent evidence highlights a non-canonical function of NINJ1 in modulating ferroptosis sensitivity through its interaction with the xCT anti-porter. NINJ1 knockdown stabilizes xCT, enhancing cellular levels of GSH and CoA, both essential for ferroptosis protection. Conversely, NINJ1 overexpression destabilizes xCT, reducing GSH and CoA levels, thereby sensitizing cells to ferroptosis triggered by xCT inhibitors [16]. This regulatory role positions NINJ1 as a potential therapeutic target for selectively enhancing ferroptosis sensitivity in cancer cells, especially those reliant on xCT-mediated metabolic pathways.

The context-dependent roles of NINJ1, which vary across tumor types, genetic mutations (e.g., p53 status), and microenvironmental conditions, demand further investigation. For instance, the combination of lipid-lowering drugs, such as bezafibrate, with ferroptosis inducers has already shown promise in overcoming the protective effects of lipid-rich microenvironments, including ascitic fluid and lymphatic niches [131,134]. Therefore, understanding how NINJ1 modulates ferroptosis sensitivity in metastatic niches, including lipid-rich environments like ascitic fluid or antioxidant-rich lymphatic systems, could provide new insights for targeting metastasis. Additionally, its interaction with ferroptosis regulators, such as xCT and p53, along with its involvement in inflammation-related diseases, further emphasize its multifaceted biological impact.

Despite this exciting progress, much remains to be investigated to fully realize the therapeutic potential of these understandings. Future research should prioritize developing selective NINJ1 activators or inhibitors to harness its therapeutic potential. Given the context- and tumor-specific role of NINJ1, NINJ1 activators could be used to target cancer cells resistant to ferroptosis while minimizing damage to normal tissues (Figure 4B,G). Conversely, NINJ1 inhibitors could be applied in cancer cells that rely on NINJ1 for proliferation (Figure 4F). Furthermore, integrating NINJ1-activating strategies with existing treatments, including immunotherapies and ferroptosis inducers, may offer synergistic benefits. Leveraging NINJ1 as a biomarker to predict ferroptosis sensitivity and treatment response could help refine therapeutic approaches and support precision medicine initiatives. Lastly, understanding the upstream signaling pathways and structural mechanisms that govern NINJ1 activation—such as its oligomerization and interaction with other membrane proteins and specific lipid moieties—could open new avenues for precise and individualized intervention. By bridging insights from basic research to clinical application, NINJ1-targeted therapies can potentially transform treatment paradigms for cancer and inflammatory diseases, offering hope for improved patient outcomes.

## Figures and Tables

**Table 1:** Potential therapeutic implications of NINJ1.

NINJ1 Function	Disease/Cell Type	Mechanism	Ref.
**Cancer**			
Promote tumor migration/invasion	Advanced prostate cancer	Enhance cell motility	[9]
Enhance tumor radiotherapy resistance	HUVECs	Ionizing radiation upregulates NINJ1 via a p53-dependent mechanism	[10]
Depend on p53 genetic status	Cells with a mutant p53	NINJ1 deficiency promotes cell proliferation and migration	[11]
	Cells with a WT p53	NINJ1 deficiency suppresses cell growth and migration	
Tumor promotion	Non-small cell lung cancer	via ligand-independent activation of LRP6/β-catenin signaling	[12]
	Colitis-associated colorectal cancer	NINJ1 deficiency reduces inflammation and tumor development	[13]
Serve as a diagnostic biomarker	Hepatocellular carcinoma and acute lymphoblastic B-cell leukemia	NINJ1 is upregulated	[14,15]
Tumor suppression	Fibrosarcoma and triple-negative breast cancer	Enhance cancer cell sensitivity to xCT inhibitor-induced ferroptosis by reducing xCT levels and activity	[16]
	Lung cancer	Suppress lung cancer cell motility via inhibition of IL-6/STAT3 signaling	[17]
	Colitis-mediated colon cancer	Inhibit macrophage migration and invasion via repression of FAK signaling	[18]
	Human hepatoma cells	Induce p21 expression and cellular senescence independently of p53	[19]
Serve as a prognostic biomarker	Ovarian cancer and urothelial bladder cancer	Correlates with slower progression and improved outcomes	[20,21]
**Nervous system**			
Promote neural development	Neuropsychiatric disorder	Mice lacking NINJ1 display repetitive and anxiety behaviors	[2]
**Vascular homeostasis**			
NINJ1 inhibition restores erectile function	Diabetic and cavernous nerve injury mouse models	NINJ1 inhibition promotes penile angiogenesis and neural regeneration via Ang1 and Ang2 signaling	[22,23]
**Bone homeostasis**			
Promote the survival of prefusion osteoclasts	Osteoclasts	Suppress caspase-9-dependent intrinsic apoptosis	[24]
**Muscle homeostasis**			
Contribute to myocyte growth and differentiation	Cardiac hypertrophy	These effects are mainly mediated by N-glycosylated NINJ1	[25]
**Inflammation**			
Promote inflammation	Experimental autoimmune encephalomyelitis and postischemic brain	Facilitate immune cell migration to sites of inflammation	[26,27,28,29,30]
	Septic mouse model	via mediating leukocyte migration and TLR4 signaling activation	[31]
	Colitis and pulmonary fibrosis	Promote macrophage activation and pro-inflammatory factor secretion	[32,33]
	Ischemic stroke	N-NAM-targeting peptides block neutrophil infiltration and reduced inflammation	[34]
	Liver injury and septic mouse model, severe acute pancreatitis, and oxalate nephropathy	Inhibit NINJ1 polymerization, leading to reduced PMR and inflammatory content release	[35,36,37,38]
	HEK293T and Raw264.7 cells	sNINJ1 acts as a chemoattractant due to its structural similarity to chemokines	[6]
Anti-inflammation	Atherosclerosis	sNINJ1-mimetic peptides inhibit monocyte recruitment and restrain macrophage differentiation	[8]

## Abbreviations

AA	Arachidonic acid
ACSL4	Acyl-CoA synthetase long chain family member 4
AdA	Adrenic acid
AIFM2	Apoptosis-inducing factor mitochondria-associated 2
Ang	Angiopoietin
AR	Androgen receptor
BMDM	Bone marrow-derived macrophage
CoA	Coenzyme A
CoQ10	Coenzyme Q10 or ubiquinone
CoQ10H2	Ubiquinol
CTCs	Circulating tumor cells
DAMP	Damage-associated molecular pattern
DDR2	Discoidin domain receptor tyrosine kinase 2
DMT1	Divalent metal transporter 1
ECM	Extracellular matrix
EMT	Epithelial-to-mesenchymal transition
ER	Estrogen receptor
ESCRT-III	Endosomal sorting complexes required for transport III
FIN	Ferroptosis-inducing agent
FPN	Ferroportin
FPP	Farnesyl diphosphate
FSP1	Ferroptosis suppressor protein 1
FTH1	Ferritin heavy chain
FTL	Ferritin light chain
GCL	Glutamate–cysteine ligase
GGPP	Geranylgeranyl diphosphate
GPX4	Glutathione peroxidase 4
GSR	Glutathione reductase
GSH	Glutathione (reduced)
GSSG	Glutathione disulfide (oxidized)
HIF	Hypoxia-inducible transcription factor
HMGB1	High mobility group box 1
HMGCR	3-hydroxy-3-methylglutaryl-CoA reductase
HMGCS2	3-hydroxy-3-methylglutaryl-CoA synthase 2
4-HNE	4-hydroxynonenal
HUVEC	Human umbilical vein endothelial cell
ICD	Immunogenic cell death
IFNγ	Interferon-gamma
IKE	Imidazole ketone erastin
IPP	Isopentenyl pyrophosphate
LA	Linoleic acid
LDH	Lactate dehydrogenase
LIP	Labile iron pool
LOX	Lipoxygenase
LPCAT3	Lysophosphatidylcholine acyltransferase 3
LPS	Lipopolysaccharides
MBOAT1/2	Membrane-bound O-acyltransferase domain containing 1/2
MDA	Malondialdehyde
MEF	Murine embryonic fibroblast
MESH1	Metazoan SpoT homolog 1
MMP9	Matrix metalloproteinase 9
MTDH	Metadherin
MUFA	Monounsaturated fatty acid
NAD	Nicotinamide adenine dinucleotide
NADK	NAD kinase
NADPH	Nicotinamide adenine dinucleotide phosphate
NINJ1	Ninjurin 1
N-NAM	N-terminal adhesion motif of NINJ1
NOX	NADPH oxidase
NSCLC	Non-small cell lung cancer
PC	Phosphatidylcholine
PE	Phosphatidylethanolamine
PGE2	Prostaglandin E2
PHGPX4	Phospholipid hydroperoxide GPX
PL	Phospholipid
PLOH	Lipid alcohol
PLOOH	Lipid hydroperoxide
PMR	Plasma membrane rupture
POR	Cytochrome P450 oxidoreductase
PPARγ	Peroxisome proliferator-activated receptor gamma
PUFA	Polyunsaturated fatty acid
ROS	Reactive oxygen species
RSL3	RAS-selective lethal 3
SAS	Sulfasalazine
sNINJ1	a soluble form of NINJ1
SLC3A2	Solute carrier family 3 member 2
SLC7A11	Solute carrier family 7 member 11
SQLE	Squalene epoxidase
SQS	Squalene synthase
SREBP2	Sterol regulatory element binding transcription factor 2
STEAP3	Six-transmembrane epithelial antigen of prostate 3
TAZ	Transcriptional coactivator with PDZ-binding motif
Tf	Transferrin
TfR	Transferrin receptor
TLR4	Toll-like receptor 4
TME	Tumor microenvironment
TRP	Transient receptor potential
TXNRD2	Thioredoxin reductase 2
VKH2	Vitamin K hydroquinone
Wnt7b	Wnt family member 7b
YAP	Yes-associated protein 1
ZEB1	Zinc finger E-box binding homeobox 1
